# Roles and Priorities of Payers, Hospitals, and Home Health Agencies in Post-Acute Home Health Care

**DOI:** 10.1093/geroni/igaf122.3500

**Published:** 2025-12-31

**Authors:** Emily Gadbois, Marguerite Daus, Jennifer Bunker, Jamie Smith, Christine Jones, Kali Thomas

**Affiliations:** Brown University School of Public Health, Providence, Rhode Island, United States; University of Colorado Anschutz Medical Campus, Aurora, Colorado, United States; Johns Hopkins University, Baltimore, Maryland, United States; Widener University, Philadelphia, Pennsylvania, United States; University of Colorado School of Medicine, Aurora, Colorado, United States; Johns Hopkins University, Baltimore, Maryland, United States

## Abstract

Home health care (HHC) provides skilled nursing, therapies, and aide services to help older adults - many of whom are recently discharged from the hospital - regain function, restore health, and maintain independence in the community. The provision of post-acute HHC is influenced by multiple players: Medicare Advantage (MA) plans, post-acute care management companies (CMCs), hospitals, home health agencies (HHAs), and patients and families. To understand the roles and priorities of these players in organizing and delivering post-acute HHC, we conducted in-depth, semi-structured interviews with 44 leaders of MA plans, post-acute CMCs, and HHAs across the country. Analyzed interviews revealed that most MA plans and CMCs agreed that home is the ideal setting and that institutional care should be avoided whenever possible; however, MA plans and CMCs varied in their involvement in post-acute home health referrals, and HHAs tended to view MA and CMC involvement as adding burden to the referral process (Theme 1). MA plan, CMC, and HHA leaders viewed the role of the hospital as to select the appropriate post-acute care setting; however, interview participants reported that these actions can be influenced by relationships with MA plans and HHAs (Theme 2). HHAs were reported to try to balance clinical complexity, staffing, patient location, and payer when accepting referrals, but had relatively limited control in the post-acute referral process (Theme 3). Insights from this work can help policymakers, payers, HHAs, hospitals, and patients understand the varied roles and shared incentives in coordinating post-acute HHC for vulnerable older adults.

